# Managing daily surgery schedules in a teaching hospital: a mixed-integer optimization approach

**DOI:** 10.1186/1472-6963-14-464

**Published:** 2014-10-15

**Authors:** Raul Pulido, Adrian M Aguirre, Miguel Ortega-Mier, Álvaro García-Sánchez, Carlos A Méndez

**Affiliations:** Escuela Técnica Superior de Ing. Industriales (ETSII-UPM), José Gutierrez Abascal 2, 28006 Madrid, Spain; INTEC (UNL-CONICET), Güemes 3450, 3000 Santa Fe, Argentina; Politecnico di Milano, Via Lambruschini 4/b, 20156 Milan, Italy

**Keywords:** Scheduling, MILP, Simulation, Operation rooms, Teaching hospital

## Abstract

**Background:**

This study examined the daily surgical scheduling problem in a teaching hospital. This problem relates to the use of multiple operating rooms and different types of surgeons in a typical surgical day with deterministic operation durations (preincision, incision, and postincision times). Teaching hospitals play a key role in the health-care system; however, existing models assume that the duration of surgery is independent of the surgeon’s skills. This problem has not been properly addressed in other studies. We analyze the case of a Spanish public hospital, in which continuous pressures and budgeting reductions entail the more efficient use of resources.

**Methods:**

To obtain an optimal solution for this problem, we developed a mixed-integer programming model and user-friendly interface that facilitate the scheduling of planned operations for the following surgical day. We also implemented a simulation model to assist the evaluation of different dispatching policies for surgeries and surgeons. The typical aspects we took into account were the type of surgeon, potential overtime, idling time of surgeons, and the use of operating rooms.

**Results:**

It is necessary to consider the expertise of a given surgeon when formulating a schedule: such skill can decrease the probability of delays that could affect subsequent surgeries or cause cancellation of the final surgery. We obtained optimal solutions for a set of given instances, which we obtained through surgical information related to acceptable times collected from a Spanish public hospital.

**Conclusions:**

We developed a computer-aided framework with a user-friendly interface for use by a surgical manager that presents a 3-D simulation of the problem. Additionally, we obtained an efficient formulation for this complex problem. However, the spread of this kind of operation research in Spanish public health hospitals will take a long time since there is a lack of knowledge of the beneficial techniques and possibilities that operational research can offer for the health-care system.

**Electronic supplementary material:**

The online version of this article (doi:10.1186/1472-6963-14-464) contains supplementary material, which is available to authorized users.

## Background

Teaching hospitals play a key role in the majority of health-care systems: these institutions provide medical attention to the community and train future health professionals. Several studies have identified operating rooms (ORs) as a hospital’s largest cost area [1, 2]. Optimizing ORs is difficult since many constraints need to be considered, and solving this issue within a reasonable time is difficult [3]. Improvements made in the scheduling of an OR lead to enhanced cost efficiency and better patient service [4].

In this situation, the objective is to determine the optimal assignment of ORs and surgeons to each operation in a daily base; consequently, it is necessary to find the best sequence of operations for each surgeon with the goal of minimizing the total surgical cost resulting from an OR’s underuse or overuse and from the surgeons’ waiting times. Here, we will assume that a set of surgeries is known 24 hours in advance of the operations and that the number of available ORs and surgeons is also known. All the planned operations have to be performed on the surgical day. The tasks that have to be performed in the surgery are divided into the following:

- TP: preparation time (preincision),
- TS: surgery time (incision), and
- TC: cleanup time (postincision).

The OR staff has to support a surgery during the preparation, the operation itself, and in the cleanup. However, surgeons are required to be present only during the operation itself until the completion of the incision. Thus, surgeons could perform an operation in a different OR immediately after finishing the previous surgery. One example of this type of decision making process is found in a Teaching hospital in Toledo, where we started describing the actual decision making process and then a computer aid decision would be introduced.

### Conventional decision-making process

This description of the conventional decision-making process is based on interviews conducted at a teaching hospital in Toledo, Spain. After a negotiation among the chief of physicians and the head of the different medical sections, ORs are assigned to each section.

For example, two ORs may be available for urology from Monday to Friday, though an additional OR is available on Wednesday. Despite most of its ORs are able to handle all medical services, in order to unnecessary changes of specific instruments required for particular medical services, the medical services use the same ORs in a weekly base.

Every day the head of the service decides which patients on the waiting list will undergo surgery and in what order and which surgeons will perform the operations. At the Toledo hospital, the head of each medical service made manually this difficult decision. Finally, a secretary puts all these details into the hospital’s computer system; the information is sent to the hospital’s reception office so that all the necessary procedures and preparations for surgery may begin. If for any reason, the patient is unable to undergo the operation, it has to be rescheduled.

### Teaching hospitals

Health care systems relies on teaching hospitals to train future health professionals, conduct medical research, fulfill part of the patient-care needs, and sometimes offer services not available in other facilities [5]. Various studies have found that resident doctors take longer to perform a surgical operation than experienced doctors. Becoming a properly trained surgeon requires that resident doctors work and study for 4–5 years, depending on their intended medical specialty, and during this time they carry out different types of surgery. The scheduling of surgery being performed in teaching hospitals has not been properly addressed in the literature [6].

Two typical differences between a normal hospital and a teaching hospital is that in normal hospital the surgery is pre-assigned to a surgeon according with any decision criteria such as the one that diagnostic the problem or the one chosen by the patients. Then, it is just necessary coordinating the use of the operation rooms. The second main difference is that the surgery duration depends on the surgical team assigned, this add the decision to evaluate which surgeon is better to assign to each surgery and where to perform the surgery.

A teaching hospital may be considered as a particular case of a normal hospital where the different resources (surgeons) may take different times, according to their experience, and there is no pre - assignment of surgeons to surgeries.

Some normal hospital algorithms have to make some assumption and preassign surgeries to surgeons to deal with teaching hospital problems. Example of this is Fei [2] that pre assign the surgical case to be treated for different surgeons (or, more generally, surgery groups) and the duration of the surgery is independent of the surgeon. Jebali [7] allows the model to assign surgeon to perform an operation but do not make any distinction with the surgeons. Other algorithms like Kharraja [8] defines a block scheduling where each surgeon request block of time to perform surgeries.

### Literature review

A literature review can make different classifications according to patient characteristics (elective or not elective), performance measures, decision delineation (date, time, room, or capacity), research methodology, and considerations of uncertainty and applicability [9]. The schedule for ORs is usually done in an intuitive manner by the OR actors; thus, introducing optimization techniques will require a cultural change because it may restrict the authority of some of those individuals [7].

Many studies have addressed different aspects of the topic of optimization techniques from various points of view with regard to the decision-making process in OR scheduling. There are different classifications of the problems in this area. One of the most important issues is decision delineation. No agreement has been reached about classifying the decisions made regarding surgery and its scheduling. Since the boundaries are unclear, various papers have addressed different parts of the decision-making process [10].

A literature review conducted by Guerreiro [11] made an interesting classification of hierarchical decision levels. *Strategic* is when OR times are assigned among different surgical services. This is also known as the “case mix planning problem”. *Tactical* involves the development of a master surgical schedule (MSS). An MSS is a schedule that defines the number and type of available ORs. There is also the *operational* type, which is concerned with the scheduling of elective patients on a daily basis after an MSS has been developed.

The strategic level of decision making is generally performed following annual negotiations between the hospital manager and the head of surgical services. This part of the decision-making process is beyond the scope of the present study. Accordingly, in terms of the hierarchical decision-level classification, this study tackles a combination of tactical and operational problems.

Another important literature review—one by Cardoen [9]—deliberately avoids these classification levels since they lack clear definitions. Cardoen [9] suggests creating a classification according to the type (date, time, room, and capacity) and level (discipline, surgeon, and patient) of decision being made. The type of decision in question could be the assignment date on which surgery will be performed, the time indications, the operating surgeon, the OR, and the allocation capacity. In this study, we will take all these elements into consideration—except for date.

With an open scheduling strategy, surgeons submit a request for OR time, and a detailed schedule is generated prior to the day of surgery. This procedure is common, for example, in neurosurgical operations, where the patient list is known only 24 hours before the day of surgery. This flexible scheme avoids unfilled blocks in the working day [12]. In the present study, we will focus on the deterministic daily scheduling problem in ORs under an open scheduling strategy.

The performance measures examined in the literature are the following: waiting time (patient, surgeon, and throughput); utilization, underutilization/under time (OR, ward, and intensive care unit); overutilization or overtime (OR and ward); general (OR and ward); leveling (OR, ward, post anesthesia care unit, holding area, and patient volume); makespan; patient deferral; financial measurements; and surgeons’ preference [9].

The aim of the present study was to develop a generic deterministic model for dealing with the daily scheduling of a set of surgeries in a teaching hospital in a reasonable time. The surgeries can be performed in a given number of ORs by different types of surgeons. We consider most of the problems encountered in the OR’s daily operations. We evaluated the proposed approach using real data from a Spanish hospital, a friendly and efficient computer aided tool and a simulation software.

The paper is organized as follows. The next section describes the actual decision-making process and the proposed approach used to address the surgical scheduling problem in a typical teaching hospital. That will be followed by the Results and Discussion. Finally, the Conclusions will be presented.

## Methods

Operation research techniques have helped health-care managers optimize their operations. We will address this issue using a mixed-integer programming model (MILP) and a user-friendly interface; these will allow the scheduling for surgeries planned the following day. Additionally, we will implement a simulation model to facilitate the evaluation of different dispatching policies related to surgical operations and surgeons. AMILP solution has previously been developed for a similar problem [13];however, that did not exploit the real strength of general-precedence concepts and did not preassignan operation to a surgeon or use different types of surgeons.

The MILP model was created using AIMMS 3.14 [14] and was solved with the standard solver Gurobi Optimization 5.5; it was simulated with Enterprise Dynamics 8.01 by In control. As noted above, the model presented is a generic one as applied to one Spanish teaching hospital. If you want to know more about the Spanish health system please refer to the Appendix. In the remainder of this section, we present a 3D simulation of the different dispatching policies, which will be followed by the MILP formulation.

### Objective function

Some studies have found that OR performance measures, such as utilization, overtime, and on-time performance, may be used as achievable targets at most hospitals [15]. Denton [16] highlights how despite the tightness of surgical schedules, it is possible to achieve a balance among the three competing criteria of surgeon waiting, OR staff idling, and overtime costs. The objective function minimizes the sum of these three costs.

- *Surgeon waiting cost.* Since the surgeon is a very expensive resource, decreasing the surgeon’s waiting time has been the subject of many papers [12, 16–18]. This factor has to take into consideration the minimum waiting time a surgeon needs between operations (Pause Time).
- *OR waiting cost* (underutilization). OR idling is the direct cost associated with having an OR vacant, with no surgical activity being performed [9, 18–23].
- *Overtime cost*. Late starts result in direct costs associated with overtime staffing when the surgery finishes later than the end of the appropriate shift [8, 12, 17, 22, 23].

The OR staff works in a normal shift of 7 hours (T = 420 minutes). Accordingly, overtime needs to be considered if the OR staff has to work beyond the normal shift length, T. For simplicity, all the patients are ready to start the surgical procedure when the OR is ready. Three main costs are taken into account (see Table 1): (a) the cost per hour of OR idling time (vacant time cost); (b) the cost per hour of OR overtime (overtime cost); and (c) the cost per hour of surgeon waiting time (waiting time cost).

**Table 1:** **Estimated hourly cost**

ORs’ vacant cost	Surgeons’ waiting cost	ORs’ overtime cost
CV	CW	CO
€ 900	€ 700	€ 1500

### Assumptions

**Figure 1:**
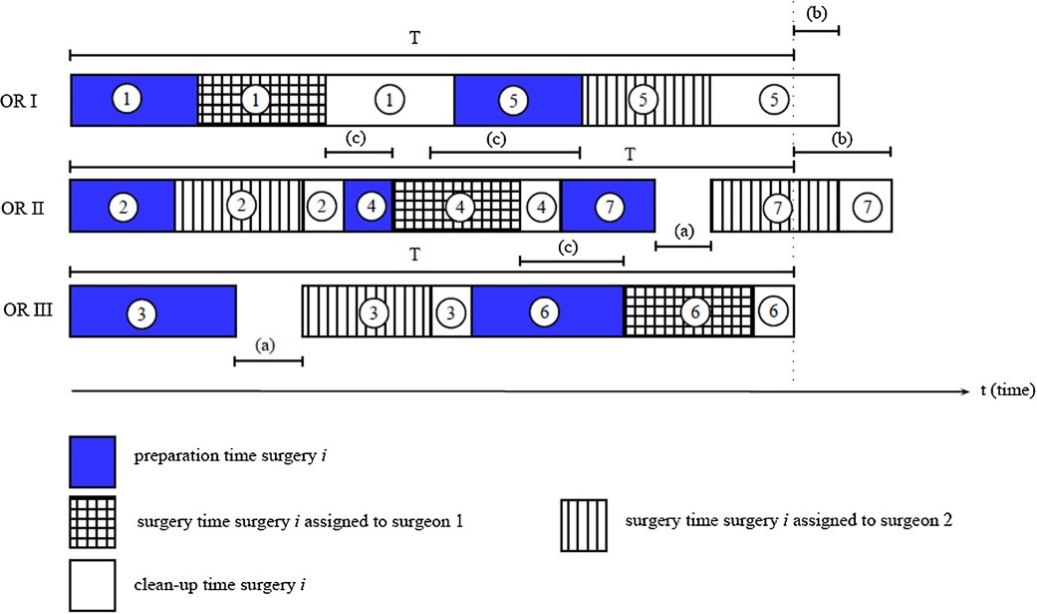
**Scheduling of a given surgical day.** Where (1–7) are the surgeries performed in three ORs with two surgeons. And (a) is the OR idle time, (b) is the over time, and (c) is the waiting time of the surgeons.

We assume that a set of ORs and surgeons are available each day. Additionally, we stipulate that only surgeon 1 in OR 1 can perform surgery D, which is an extremely complex operation, and that surgery A should be performed by a resident (surgeon k = 2). The remaining surgeries can be performed in any OR by any surgeon. The ORs can operate in parallel.Figure 1 presents a simple example of seven surgeries scheduled in three ORs with two surgeons. The first idle time cost (a) is incurred when the patient has to wait for surgeon 2. OR1 and OR2 generate extra time cost (b). Surgeon 1 generates waiting cost when the surgeon finishes surgery 1 and has to wait to start surgery 4. The vacant time is the time between surgeries where the surgeon cannot perform other activities since the surgeon is wearing surgical uniform.

### Data accessibility

We looked for available public data to test our model, but the majority of the papers dealing with OR scheduling do not present a complete dataset. We used data from the waiting list of the urology department of the general hospital in Toledo, mentioned above, to test the model. That information included an estimate of the duration of the surgery.

We experimented with six instances, each consisting of five to nine surgeries. Table 2 contains the preparation time, the incision time for each surgeon, and the cleanup time. Each surgeon had different surgical times according to their expertise, which allowed them to perform an operation faster or slower. Surgeon (k = 1) was the fastest surgeon, surgeon 2 the slowest, and surgeon 3 intermediate. Each instance represents different types of working days, with two ORs and three surgeons being available. For example, instance 1 represents the smallest instance, in which only five surgeries have to be performed, and instance 6 represents the largest instance, in which nine surgeries have to be performed (see Table 3). To test the model, we ran it on a day with the following availability: three ORs and two groups of surgeons—one without residents (*k* = 1) and the other with residents (*k* = 2).

**Table 2:** **Surgery durations (min.)**

Surgery type (i)	A	B	C	D	E	F	G	H
**TP**	15	20	15	20	25	30	35	40
**TS**_**k=1**_	20	35	40	45	85	130	190	220
**TS**_**k=2**_	30	53	60	n.a.	128	195	285	330
**TS**_**k=3**_	25	44	50	n.a.	106	163	238	275
**TC**	10	20	35	40	40	50	50	60

**Table 3:** **Surgical day instances with several ORs and surgeons**

#Instance	#ORs	***#k***	#Surgeries	S1	S2	S3	S4	S5	S6	S7	S8	S9
**1**	2	1	6	A	B	C	D	E	E			
**2**	3	2	5	E	E	D	F	G				
**3**	3	2	6	C	D	D	E	F	H			
**4**	3	2	7	B	B	C	D	E	G	G		
**5**	3	2	8	A	B	B	C	D	E	F	G	
**6**	4	3	9	A	B	C	D	E	E	F	G	H

Spanish hospitals usually operate from 8:00 a.m. to 3:00 p.m. (T = 420 minutes). Extra time is possible only if a request is made for this during the day. Thus, it is important to know when additional time will be required, and it cannot exceed 2 hours. If any delays occur aside from the approved extra time, the surgeon and other staff need to finish the surgery without additional payment. Therefore, if the anesthesiologist or surgeon realizes that a surgery will not be completed on time, they usually prefer to cancel the surgery and reschedule it before it begins.

### Simulation model

We used a simulation to evaluate different solutions without any disturbance on the hospital’s operations [24]. We built the simulation model using the Enterprise Dynamics discrete-event simulation tool, which emulates different dispatching policies of surgeries and surgeons.

We set different strategies for the dispatch of surgeries such as ordering (ascending or descending) them according to the duration of a given surgery time (TS). When two surgeons were idle, we selected either the faster or slower surgeon to perform the surgery [18].

### MILP problem formulation

In this section, we begin by introducing the notation needed to formulate the problem (see Table 4). Thereafter, we present the MILP for the OR scheduling.

**Table 4:** **Indexes, parameters, and variable sets**

Sets	
*S*	Set of surgeries *s* to be scheduled in a surgical day
*S*_*k*_	Subset of surgeries *(S)* that can be performed by surgeon *k*
*S*_*r*_	Subset of surgeries *(S)* that can be performed in room *r*
*R*	Set of operating rooms *r*
*K*	Set of surgeons *k*
**Parameters**	
*TP*_*s*_	Preparation time (preincision time) of the surgery *s*
*TS*_*sk*_	Surgery time (incision time) of the surgery *s* by surgeon *k*
*TC*_*s*_	Cleanup time (postincision time) of the surgery *s*
*CV*	Cost per minute of having an OR vacant
*CW*	Cost per minute of having the surgeon waiting
*CO*	Cost per minute of using an OR beyond the normal shift length *T*
*T*	Shift length
*PT*	Pause between surgeries done by the same surgeon
*MOT*	Maximum overtime
*MaxS*	Maximum number of surgeries performed by a surgeon
*M*	A large scalar value
**Variables**	
*x*_*sr*_	Binary variable; 1 if surgery *s*∈ *S* is done in room *r*∈ *R*, 0 otherwise
*y*_*ss’k*_	Binary variable; 1 if *s*∈*S* precedes *s*'∈*S*and is done by the same surgeon *k*∈*K*, 0 otherwise
*z*_*ss’kk’*_	Binary variable; 1 if *s* precede *s*'∈*S* and it is done by different surgeon *k* and *k*'∈*K*, 0 otherwise
*q*_*sk*_	Binary variable; 1 if surgery *s*∈ *S* is done by surgeon *k*∈ *K*, 0 otherwise
*msR*_*r*_	Non negative variable equal to the make span of room *r*∈ *R*
*msS*_*k*_	Non negative variable equal to the make span of surgeon *k*∈ *K*
*ts*_*s*_	Non negative variable equal to the start time of the surgery *s*∈*S*
*tsS*_*k*_	Non negative variable equal to the start time of the surgeon *k*∈ *K*
*vt*	Non negative variable equal to the vacant time
*ot*_*r*_	Non negative variable equal to the overtime of room *r*∈ *R*
*wt*_*k*_	Non negative variable equal to the waiting time of a surgeon *k*∈ *K*
*tc*	Total cost

Most studies [2, 12, 17] on this topic make the assumption that the surgeons for each patient are already known. However, the problem of assigning surgeons, residents for each operation in several ORs does not appear to have been addressed in OR planning and scheduling [25]. To the best of our knowledge, no models have involved different types of operations in multiple ORs with different types of surgeons. This study presents a detailed scheduling scheme for different surgery cases in multipurpose ORs with multiple types of surgeons. Based on the principal ideas of general-precedence concepts [26], we formulated a MILP model for the scheduling of multiple surgery types in multiple ORs with several available surgeons.

We present a MILP model for this particular problem with the aim of minimizing the total surgical cost denoted by the overtime cost (*CO*), vacant time cost (*CV*), and waiting time cost (*CW*) in equation (1). Equations (2) and (3) guarantee that all surgeries are performed in only one OR by just one surgeon. The sequencing and timing constraints in the same OR and also by the same surgeon are presented in equations (4) and (5) and equations (6) and (7), respectively.

The binary variable *z*_*ss’kk’*_ is introduced to consider the sequencing and timing decisions of operations performed by different surgeons but in the same OR, as shown in equations (8) and (9). Equations (10) and (11) are provided to estimate the completion time both in the ORs (makespan of the rooms) and by the surgeons (makespan of the surgeons). Equations (12) and (13) determine the initial time of each operation and surgeon in the system. Additionally, overtime (*ot*) is calculated in equation (14) while equation (15) limits the amount of overtime and equation (16) limits the number of operations performed by the same surgeon. Vacant time (*vt*) and waiting time (*wt*) variables are calculated in equations (17) and (18), respectively.
123456789101112131415161718

Where


### Ethics

Given that this research is computational in nature, does not generate adverse environmental impacts neither involves human subjects and respects the existing bioethical standards.

## Results

We evaluated the performance of our approach using part of the waiting list from the urology department of the teaching hospital in Toledo. We defined six instances with different numbers and types of surgeries. The computational experiences were performed on a ASUS PC Intel Core i3-2350 M 2.30 GHz with 6 GB RAM running the solver in parallel mode with two threads under Windows 7.

### Simulation results

The results of the different strategies for various surgeries and surgeons are presented in Table 5. In the last column, the results from running 100 replications are displayed.

**Table 5:** **Costs of the different strategies for the dispatch of surgeries (euros)**

Instance	Optimal (MILP)	Faster***k***, ascending TS	Faster***k***, descending TS	Slower***k***, ascending TS	Slower***k***, descending TS	Results of 100 scenarios	
						Mean (std)	Min-Max
1	3,400	3,850	4,325	3,850	4,325	3,812 (53)	3775-3850
2	2,850	3,579	4.320	6,966	4,850	4,914 (1,277)	3,579-6,966
3	3,258	8,541	6,616	9,141	7,204	7,162 (1,337)	5,591-9,475
4	5,100	7,291	10,300	5,900	9,662	7,848 (1,104)	5,900-10,562
5	3,650	9,600	7,783	8,800	6,729	7,073(960)	5175-10,329
6	4,183	15,668	13,539	13,637	15,456	13,563 (933)	11,066-16,568

It is evident from those results that no dispatching policy was able to outperform the others. For some instances, using the faster surgeon first was better; in other instances, using the slower surgeon was advantageous. We made the same observation with the ascending or descending order. Another option was to try many random combinations to obtain a good solution. In some instances, that worked to an acceptable degree; however, with a larger number of ORs and surgeons, there was a greater difference from the optimal situation. It should be noted, though, that all of the results were far from optimal.

### MILP results

**Figure 2:**
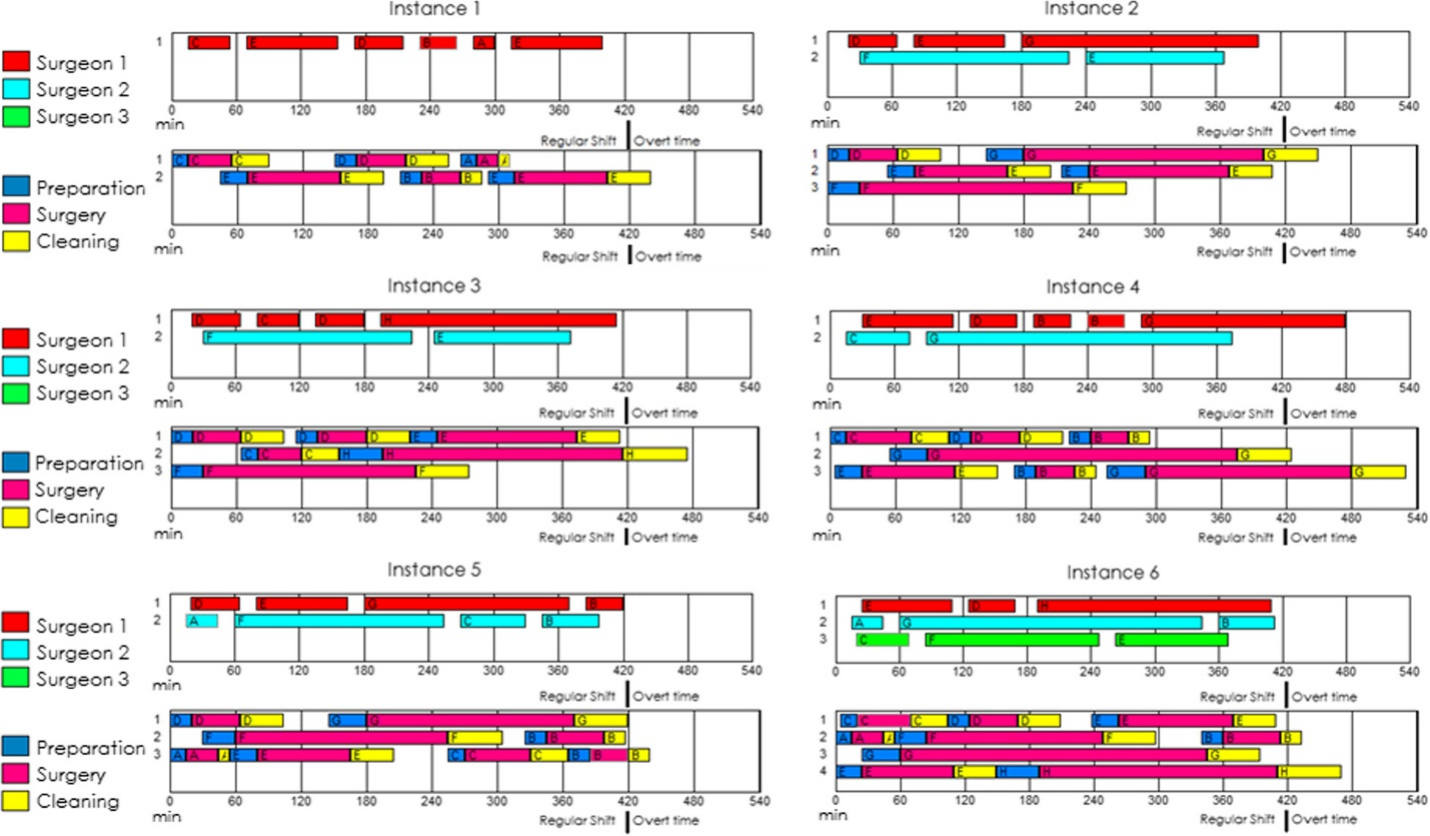
**Gantt diagram of instances 1–6.** Each panel refers to one instance. Every panel has a pair of Gantt diagrams, the upper Gantt diagram is the schedule of the surgeons and the lower Gantt diagram is the schedule of each operation room.

Table 6 presents the computational performance of each instance; Table 7 shows the detailed costs and Figure 2 displays the solution schedule in a Gantt chart. In that chart, we observed that the waiting time of the surgeons was minimized and that changing the ORs would avoid delays with the postincision time and the cleanup time for the next patient. The overtime was minimized, but it was inevitable in some situations. OR occupation also increased since in the majority of the cases as soon as one patient left, the preincision procedure began for the next.

**Table 6:** **Results of the instances**

Instance	CPU time (s)	Total cost (€)	Integer variables	Continuous variables	Constraints	Nodes	Iterations
**1**	3.8	3,400	33	15	140	10,552	36,167
**2**	3.5	2,850	65	19	357	5,812	26,076
**3**	4.7	3,258	90	20	510	4,734	21,390
**4**	140	5,100	119	21	691	224,573	948,271
**5**	456.3	3,650	152	22	900	558,804	2,984,012
**6**	1,720.1	4,183	387	28	3,013	1213370	5,627,758

**Table 7:** **Detailed cost of the instances**

Instance	1	2	3	4	5	6
**Waiting time (min)**	75	45	65	75	90	95
**Waiting time cost (euro)**	875	525	758	875	1050	1,108
**Overtime (min)**	20	30	55	115	20	63
**Overtime cost (euro)**	500	750	1,375	2,875	500	1,575
**Vacant time (min)**	135	105	75	90	140	100
**Vacant time cost (euro)**	2,025	1,575	1,125	1,350	2100	1,500
**Total cost (euro)**	3,400	2,850	3,258	5,100	3650	4,183

We did not experiment with any operations bigger than surgery type *H* (320 minutes’ duration): they would require a full day in the OR and would result in a trivial answer (one OR, one long surgery), which was not relevant to this study.

Our model was able to deal with multiple surgeons in multiple ORs. The solutions are presented in Table 6. Some of the tested instances are solved up to optimality within a few minutes—in some cases, in less than 1 minute. The model takes more time to solve the most complex instance.

The model size is also reported in Table 6 according to the number of variables and constraints; the complexity of the solution is demonstrated by the number of nodes and iterations explored. As with other similar models, the solution time for an instance with the same number of surgeries varies considerably depending on the data. This is a critical point in the solution performance: our model could obtain high-quality initial feasible results in only a few minutes, but it needed much more time to ensure the optimality of the solution found. With the general-precedence formulation, a reduced number of binary sequencing variables has been reported compared with other MILP formulations, e.g., that presented in Batun et al. [13].

Our model was refined through using pairs of constraints associated with the general-precedence formulation and appears to be much more efficient than that since it uses a unique general-precedence variable for sequencing surgeries simultaneously for both ORs and surgeons; in other formulations, the sequencing variables are proposed for each OR using surgery-specific precedence-based representation. Since the number of binary variables increases with the number of surgeries and the number of ORs is greater than the number of surgeons, our representation can significantly reduce the size of the problem [26]. As an example, using a unit-specific representation, the number of sequencing variables will be |S|*|S-1| in each OR as a result of s ≠ s’; in our formulation, the number of combinations is reduced to (|S|*|S-1|)/2 for each surgeon since s > s’ under general-precedence concepts. This is because if the sequence exists in one tuple of the constraints, it does not exist in the other.

The combinatorial sequencing problem size increases with the number of surgeries considered, as noted above. That is why it is so important to reduce the number of binary sequencing variables when solving large problems with reduced computer effort.

### Variation of the number of surgeons and ORs

This problem can be solved by varying the number of ORs and surgeons and by minimizing the total surgical cost (OR idling, surgeon waiting, and overtime). If the number of surgeons and ORs is constant, the idling time of the ORs is zero since they are never vacant. Then, the waiting time for the surgeon increases since the surgeon has to wait for both the cleanup and preparation of an OR to be completed before starting the next operation.

**Figure 3:**
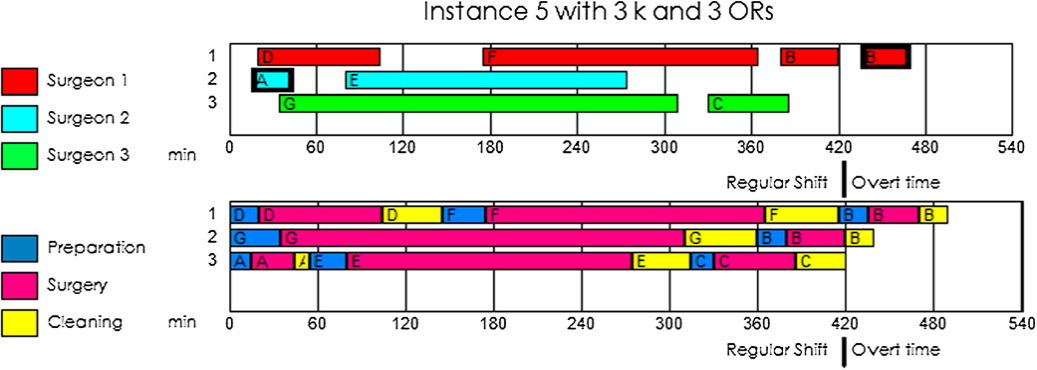
**Gantt diagram of the instance 5 using the same number of surgeons and rooms.** The upper Gantt diagram is the schedule of the surgeons and the lower Gantt diagram is the schedule of each operation room.

The final configuration will depend on the resources available on a surgical day, and the manager must decide on and evaluate the best possible option. The manager may choose to perform the surgeries with fewer surgeons or staff or use the same number of surgeons and ORs if they are available that day.In Figure 3, we present instance 5 using one extra surgeon (3 Surgeons and 3 ORs). Having the same number of surgeons as ORs meant that the cost increased from €3,650 to €4,083 (€1,800 for overtime and €2,283 for vacant time). There is no single answer as to whether it is better to have more ORs than surgeons on a given day: this situation should be evaluated for each instance with the use of the mathematical model.

### Importance of differentiated surgery times in a teaching hospital

Not all surgeons are the same. In the context of a teaching hospital, this matter becomes very important. According to Bridges et al. [6], who compared 14,452 cases in terms of operating time, that time was longer in 10,787 procedures when a resident performed the surgery rather than it being done by an experienced surgeon. As with any other process, an experienced surgeon usually works faster than a student. Some faculty surgeons have performed operations for many years, and the residents are still learning. We incorporate this feature in our model by trying to represent actual behavior at teaching hospitals. In Table 3, we assign different operation durations to different surgeons, assuming that each surgeon may perform each operation faster or slower than the projected time.

**Figure 4:**
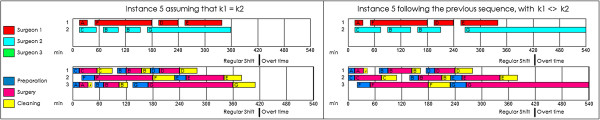
**Problems of assume that the surgeons perform the surgeries in the same time.** On the left part are the Gantt diagram (surgeons and operation rooms) of the instance 5 assuming that all the surgeons perform the surgeries in the same time. On the right part are the results of follow the previous sequence, with surgeons that perform the surgeries in different times.

The misguided assumption that all surgeons perform equally can create significant scheduling problems. This is especially important in a teaching hospital, where residents perform many operations during the surgical day. In the next example, we planned the surgical day under the false assumption that surgeon 1 and surgeon 2 (the resident) perform their operations in the same amount of time. When we reviewed the results (see Figure 4), we found that there would be no overtime: all the staff would finish early at a total cost of €1,725. In this situation, the planner could even consider including additional surgeries. However, when the surgeons followed the sequence obtained, the residents took more time, and the result was completely different: there was considerable overtime, and an increase in the total cost of up to €6,048. In the previous section (Table 6), we solved instance 5 by considering from the outset the difference among surgeons in terms of skill: the resulting cost was €3,650, which is significantly lower than €6,048.

The objective with the above example is to highlight the problem with a commonly accepted assumption when scheduling, whereby the duration of a surgery is independent of the surgeon’s skill. This could result in additional costs owing to unforeseen delays or cancellations of surgery through limitations with the extra time. For this reason, it is important to differentiate between surgeons.

### Rescheduling

**Figure 5:**
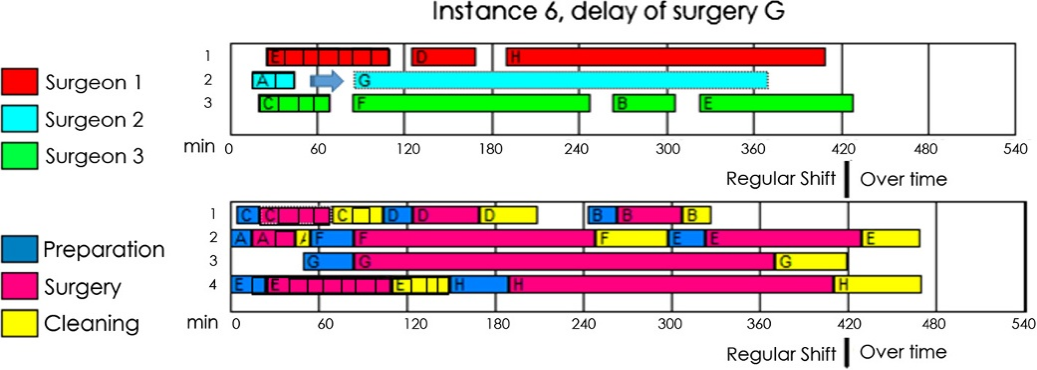
**Gantt diagrams of the rescheduling of instance 6.** The hatched surgeries (A, E, C) are fixed, and the surgery G is delayed 25 minutes. The upper Gantt diagram is the schedule of the surgeons and the lower Gantt diagram is the schedule of each operation room.

Many changes can occur in the course of a day at a hospital, such as the duration of surgeries and the starting time of those procedures. A rescheduling procedure based on fixed the variables *x*_*sr’*_*q*_*sr’*_ and *ts*_*s*_ relates to the completed surgeries and surgeries that have already begun. The start time and duration of the surgeries are modified according to new information, and scheduling can be solved up to optimality in only a few seconds using a deterministic approach. With our model, we can handle uncertainties in surgery duration and modify the schedule immediately after the occurrence of unexpected events during the surgical day. The values of the fix variables allow the determination of other values related to the general precedence for the same surgeon, for different surgeons, for different ORs, and for the same OR, thereby decreasing the overall solution time.Figure 5 presents the rescheduling in instance 6. When surgery G has a delay of 25 minutes, the algorithm fixes the variables associated with surgeries E, A, and C, and it determines the new start of surgery G. It then optimizes the remaining surgeries. The optimal situation with this example was achieved in 15 seconds. A smaller instance could be solved faster, and rescheduling based on the first surgery would take the same amount of time as normal scheduling. If we recall the optimal outcome for instance 6, presented in Figure 2, surgeon 2 should perform surgery B at OR 2 after that surgeon completes surgery G; though surgery B now becomes assigned to surgeon 3.

The rescheduling tool allows the OR planner to deal with new conditions that arise during the surgical day, implementing the required modifications to the schedule, thereby decreasing the cost impact and avoiding surgery cancellation. An example is provided in a video (see Additional file 1).

### Software interface

**Figure 6:**
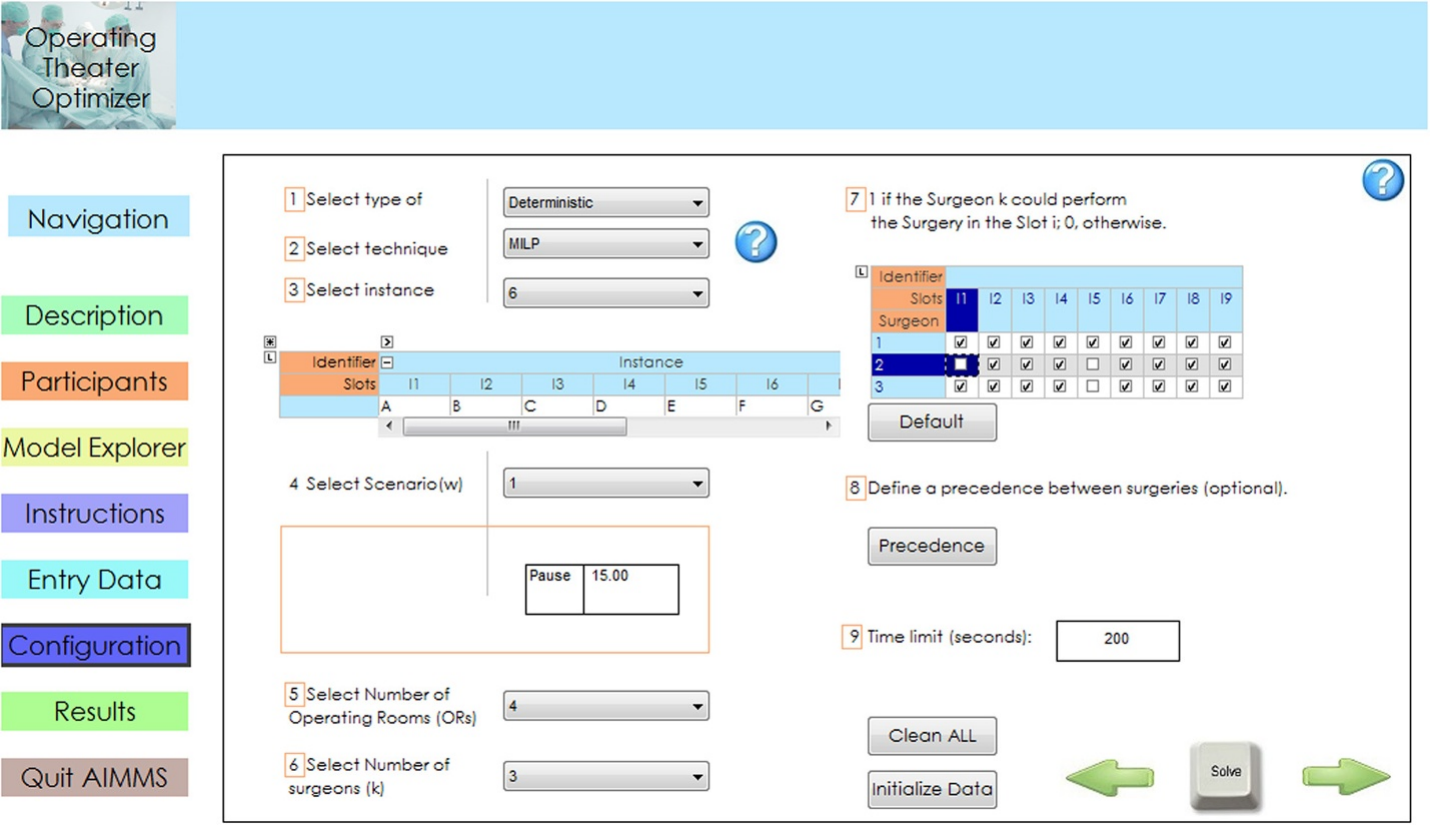
**Screenshot of the configuration page of the software interface.**

We developed a user-friendly interface in AIMMS [14] to deal with this complex optimization problem (see Figure 6). We included a guide to help users become familiar with the process. Additionally, we added a rescheduling capability, and we facilitated the changes to the experimental data. Our solution tool provides the manager with the possibility of easily changing parameters and obtaining high-quality results faster. The video presents a brief overview of the interface and its rescheduling capabilities in real time (see Additional file 1).

**Figure 7:**
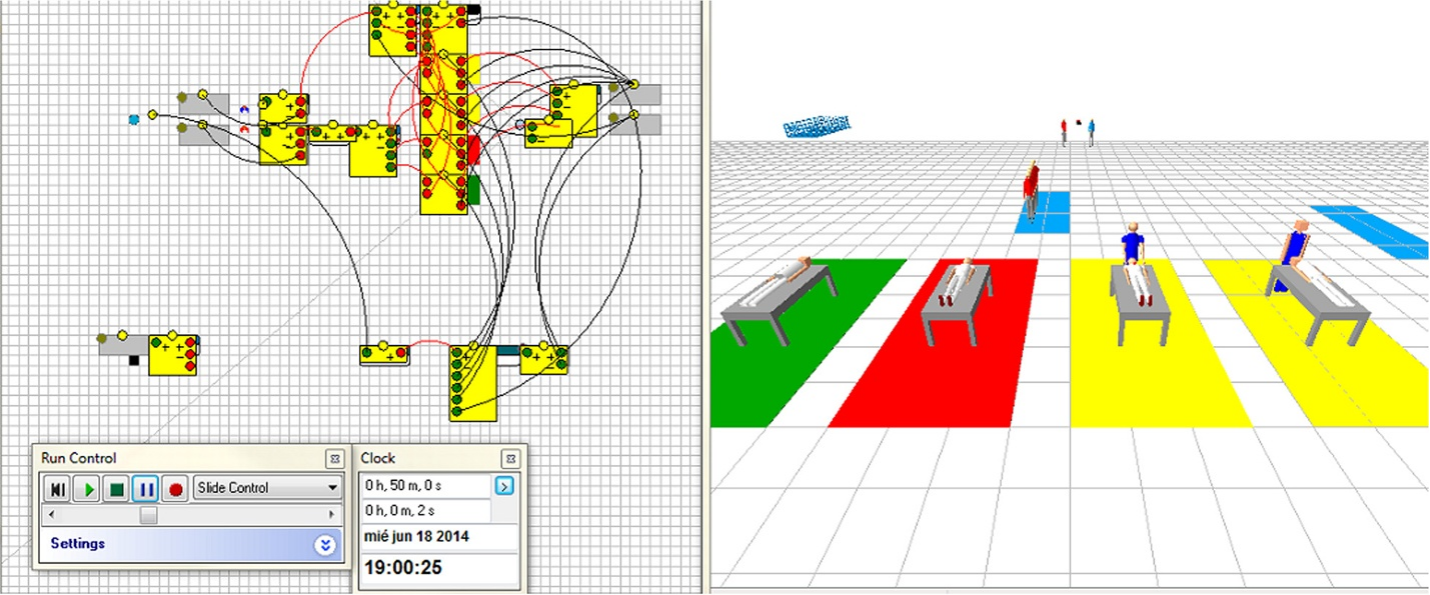
**Screenshot of the 3D simulation.** The left part is the diagram of the simulation and the right part is the 3D visualization.

We used the simulation software Enterprise Dynamics to facilitate the evaluation of the different schedules performed by various surgeons. The interface allows the planner to visualize any schedule in 3-D (see Figure 7) and to evaluate different dispatching policies for surgeons and surgeries, as indicated in Table 4. The simulation model aims to mimic the behavior of the OR, allowing the planner to easily change the parameters of the simulation and to set a predefined schedule; alternatively, the planner may introduce dispatching rules and see their effects in the accrued costs at the end of the simulation (see Additional file 2).

## Discussion

We presented the model we developed to physicians, who provided useful feedback. We took their suggestions into consideration and made some improvements to the model, such as limiting the amount of overtime available and the maximum number of operations that could be performed by a surgeon in a day. In addition, the physicians noted that it was unrealistic to perform one surgery and then immediately begin the next one. Accordingly, we added some pause time (PT) to the model; in some of the results, this time was the only waiting time cost present.

The main problem with our model arises from the fact that a surgeon sometimes moves from one OR to another OR. This procedure is currently only done in special cases at the Toledo teaching hospital, such as when the surgeons are in a hurry: the surgeons themselves prefer to perform all their scheduled operations in the same OR.

The problem with using a single surgeon for surgical operations in parallel ORs has already been examined [13, 27]. Mancilla [27] presented an interesting discussion on this topic, arriving at the conclusion that the use of parallel ORs depends on ratios: the cost ratio (cost of waiting/cost of idling) and the “setup to surgery time ratio”. The problem in limiting the mobility of surgeons is that during the cleanup time and preparation time for the next patient, the surgeon is not occupied. Therefore, there is a significant time saving if surgeons move from one OR to another, avoiding idling costs. If all the operations by one surgeon are scheduled in the same OR, a major benefit of the scheduling is lost.

### Uncertainty with times

If our model is used to develop OR schedules, having an accurate estimate of the operating time required for each surgery type is a prerequisite to its effective use. However, assessing an operation’s execution time is not easy because it depends on the patient’s pathology, which may be known only partially, and on the surgeon’s expertise [28, 29].

Since there are no historical data—either on the probabilities or distribution of the surgery duration for each patient—we followed the strategy of finding a fast, accurate solution using the time estimated by the chief of surgery. Asking the head of surgical services to provide a forecast concerning the three times (preparation, surgery, and cleanup) for each operation increases the complexity in using the system; it also increases the complexity of the model without obtaining a better solution since all the data are used for the estimate. Having an information system that stores all records relating to surgeons and patients would help increase the accuracy of such estimates.

Interesting approaches has been done in the stochastic field such as Batun [13], however, obtaining a more robust solution usually has the requirement of a high computational cost. Since the aim of this paper is to deal with the daily scheduling, using a deterministic approach to generate a good solution in a reasonable time was preferred.

Despite the use of a stochastic or deterministic model approach is advisable to periodically update the solution with the most recent data.

In case that the actual time of the surgery differs from the predicted model, the solution will be affected and it will be necessary to run the scheduling again with the new information. In order to take the best decision, for example changing the beginning of the next surgery, or the surgeon that will perform the surgery, in case of that it is needed in the rescheduling phase the minimum pause time constraint could be relaxed to minimize the impacts of the delays.

### Limitation of the model

The main limitation with our model is that we first need to define the set of surgeries that should be sequenced each day. A future developmental step would be to select from the entire waiting list which surgeries should be performed according to their urgency (time on the waiting list).

Using historical data to feed the model could help the decision maker to obtain accurate predictions of the duration of the surgeries performed by each surgeon. However, the same operation can have different times even when performed by the same surgeon because every patient is different: according to the head of the medical service, “Nobody knows what they might find when they enter the operation room”. Although we developed a general model for a teaching hospital, there are still many specific considerations that need to be studied and implemented in the final program for it to be used on a daily basis.

## Conclusions

The principal contribution of this paper is the development of an effective computer aided framework based on a mixed integer lineal programming and a simulation model for the daily schedule of the ORs of a teaching hospital managing multiples surgeries performed by different surgeons.

The MILP model is able to deal with scheduling different types of surgeries in parallel ORs and with multiple surgeons. Using this model, decisions are made on an operative level because the capacity of the resources (ORs and surgeons) and the operations that need to be performed are known 24 hours in advance.

Our model provides high-quality results within a reasonable time for the decision maker, and it allows a new schedule to be created if any circumstances change. By incorporating the advantages of model formulation, we can easily allow surgeons to specialize in only certain types of operations and deal with real-world problems without incurring additional computational costs.

The daily surgical scheduling in ORs with multiple surgeons is still a complex issue for the managing director of a hospital. Our tool was specifically designed to help managers analyze and evaluate possible profitable results within a reasonable time frame. There are several specific requirements that are significant to a manager-director that could be examined in future research toward more accurately representing real situations. Some future considerations could be the stochastic duration of the surgeries themselves, different operation durations depending on the surgeon, and the upstream and downstream resources necessary to support surgical activities, such as preoperative and postoperative actions.

These and other practical considerations provide an opportunity to continue research in this area: the promising results in terms of savings and publications represent more opportunities for operation research in health management. In addition, it is necessary to convince the decision makers about the advantage of health-care operation research; they need to know that it is worth investing their time and money in further studies of this nature even in the face of current ongoing cuts in the public health-care system.

## Appendix

### Spanish health system

Spanish hospitals provide an interesting study case since the country’s population is markedly older than most nations in Europe: 17.5% of the Spanish population is 65 years old, and the median age is 42.6 years [30]. Spain has one of the highest life expectancies in the European Union, where the average is 81.8 years; at 84.7 years, the country also has the highest female life expectancy in Europe. The implication of this aging population is that more elderly patients will need to undergo surgery and have to be placed on a surgery waiting list.

The Spanish health-care system is both public and private: according with the last report of 2013 there are 452 public hospitals and 311 private hospitals. The system incorporates 4,201 ORs for the country’s around 47 million inhabitants. Every year, 24,342 surgeons perform 4.74 million operations (1,129 surgeries per OR per year) [31].

The last official Spanish stats from 2013 counts that there are 20,721 residents doctors, from which 5,698 are working and studying to become surgical specialists in Spanish hospitals, and many of them perform surgeries every day [31].

## Electronic supplementary material

Additional file 1: **Video with a quick overview of the interface and real time solving.** (MP4 1 MB)

Additional file 2: **Video with a quick overview of the 3D simulation.** (MP4 2 MB)
